# Supplementary material for: Reliable uncertainty estimates in deep learning with efficient Metropolis-Hastings algorithms
Source: Nat Commun. 2026 Mar 17;17:2531. doi: 10.1038/s41467-026-70015-z (PMC12996495; doi:10.1038/s41467-026-70015-z)
Supplement: Supplementary file 1 — Supplementary Infomation [file 41467_2026_70015_MOESM1_ESM.pdf]

Appendix A Further Results

As this side-result played a minor role for the final results we show Phases 4 and 5 of the hyperparameter study in the appendix. Here, we tested the influence of a changed batch-size and momentum decay. Both showed only minor changes in accuracy and therefore were not evaluated further.

Appendix B Algorithms

In this section we shortly present algorithmic descriptions of the algorithms, that are used in the paper. This can show the differences and similarities as well as support reproduction.

The integrator used in the algorithms is chosen as a generic function, since it can be replaced by different suitable choices. Eventually, as in the case of the skew-symmetric integrator, a half position update is required after and before the for-loop. For the z variable we slightly abuse the set notation for a short dictionary like object, which stores elements that are accessed by their name.

Supplementary Table A1 Phase (4/5): Evaluating the influence of momentum and batch size during sampling. Baseline: $\gamma\epsilon = 0.1$, $|B| = 80$.

	RMSprop	Baseline	$\gamma\epsilon = 0.01$	$\|B\| = 400$
LeNet	✓	89.4%	89.7%	89.8%
	–	89.5%	89.4%	89.3%
ResNet	✓	86.5%	86.4%	86.7%
	–	85.8%	86.1%	86.0%

Algorithm 2 AMAGOLD algorithm with suitable integrator.

AMAGOLD($N, U, \boldsymbol{\theta}_0, t_L, \epsilon, M,$ integrator)
$\mathcal{S} = \{\}$ ▷ posterior samples
$m \sim \mathrm{N}(0, M)$ ▷ initialize momentum
$\boldsymbol{\theta} = \boldsymbol{\theta}_0$ ▷ initialize position
while $|\mathcal{S}| < N$ **do**
    $z = \{\boldsymbol{\theta}, m\}$ ▷ itore initial values of cycle
    $\rho_0 = 0$ ▷ initialize energy accumulator
    for $t = 0$ to t_L **do**
        $\tilde{m} = m$
        integrator.step($\boldsymbol{\theta}, m, \epsilon, M, \tilde{U}$)
        $\rho_{t+1} = \rho_t + \frac{1}{2}\epsilon\nabla\tilde{U}(\boldsymbol{\theta})M^{-1}(m + \tilde{m})$
    end for
    $r = \min\{1, \exp(U(\boldsymbol{\theta}_0) - U(\boldsymbol{\theta}) + \rho_{t_L})\}$
    $\nu \sim \mathrm{Unif}(0, 1)$
    if $r > \nu$ **then**
        $\mathcal{S} = \mathcal{S} \cup \{\boldsymbol{\theta}\}$ ▷ accepted!
        $z = \{\boldsymbol{\theta}, m\}$ ▷ overwrite initial values of cycle
    else
        $z(m) = -1 \cdot z(m)$ ▷ rejected!
        $\boldsymbol{\theta}, m = z(\boldsymbol{\theta}), z(m)$ ▷ reset simulation states
    end if
end while

Equally, we include an algorithmic description of the GSGHMC algorithm using the same notational guidelines.

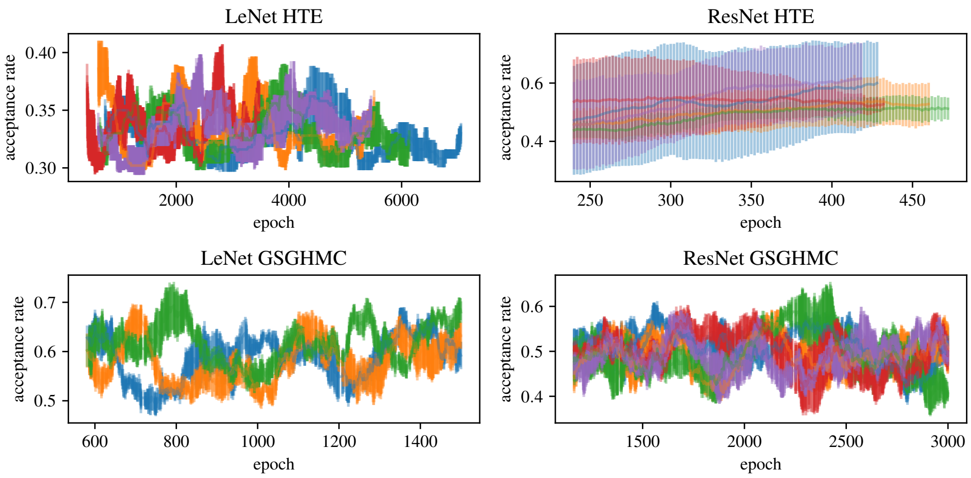

Supplementary Figure C1 Overview on the dynamics of the acceptance rates. Shown are the mean and standard deviation of the parallel chains running together in one simulation. The different colors report on different starting seeds.

Algorithm 3 GSGHMC algorithm with OBABO integrator.

GSGHMC($N, U, K, \boldsymbol{\theta}_0, t_L, \epsilon, M$, OBABO)
$\mathcal{S} = \{\}$ ▷ posterior samples
$m \sim \mathrm{N}(0, M)$ ▷ initialize momentum
$\boldsymbol{\theta} = \boldsymbol{\theta}_0$ ▷ initialize position
while $|\mathcal{S}| < N$ **do**
  for $t = 0$ to t_L **do**
    $\tau_0, \tau_p \sim \mathrm{Unif}(I_\tau)$
    $z = \{\boldsymbol{\theta}_0, m\}$ ▷ store initial values of cycle
    $m_{1/4}, m_{3/4} = \mathrm{OBABO.step}(\boldsymbol{\theta}, m, \epsilon, M, \tilde{U}_{\tau_0})$
    $r = c_n(\tilde{U}_{\tau_0}(z(\boldsymbol{\theta})) - \tilde{U}_{\tau_p}(\boldsymbol{\theta}_p))) - K(m_{3/4}) + K(m_{1/4})$
    $\nu \sim \mathrm{Unif}(0, 1)$
    if $r > \log(\nu)$ **then**
      continue
    else
      $\boldsymbol{\theta}, m = z(\boldsymbol{\theta}), z(m)$ ▷ rejected! reset state
    end if
  end for
  $\mathcal{S} = \mathcal{S} \cup \{\boldsymbol{\theta}\}$
end while

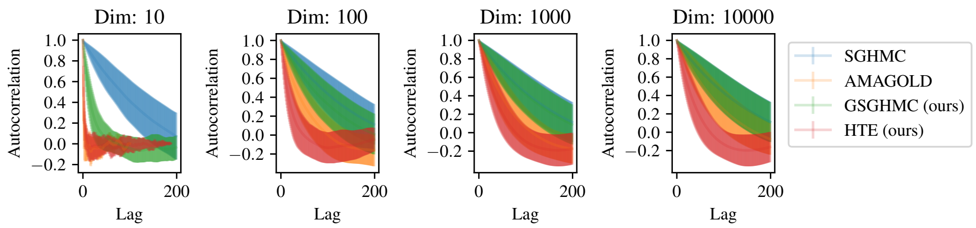

Supplementary Figure C2 The autocorrelation of samples generated by the respective methods. Reported is the shaded area of one standard deviation over the 10 random seeds.

Appendix C Acceptance Rate and Autocorrelation

As it was already discussed in the paper, the acceptance rates did not show a clear correlation to the performance metrics in the range that was interesting for us. We observe a rather random walk like behavior for most of the simulations averaging to an acceptance rate of about 50%. In the HTE case the acceptance rate showed very different dynamics comparing the ResNet and LeNet example. However, the acceptance rates did not pose the upper limit to the step-size but rather the stability of the simulation in general as it was also shown in figure 3.

The autocorrelation was highly correlated to the scores reported in figure 1. For the higher dimensions we observe the autocorrelation of GSGHMC and SGHMC to be very similar, however still showing a significant performance gap. We explain this through a controlled choice of steps that enables slightly higher step-sizes and therefore a better exploration to improve the estimation of the variance.

Appendix D Loss-Landscape

This section shows a projection of the log-likelihood on a random 2D subspace of the parameter space using techniques published in [54]. The center of the plots in figure D3 is a minimum found with a regular deterministic neural network training. The color ranges from green for small to yellow for large loss values in between 0.1 and 10.

The plots can yield some intuition about the results presented in the paper. We observe many minima, which are close by in the ResNet case, while the LeNet example seems to have large minima, which are very unlikeli to escape with an sampling algorithm. We believe the HTE method to effectively explore many minima of this distribution, while the SGHMC methods rather carefully explore a local region. That seems to improve the calibration, but misses the improvements in accuracy we could see using the HTE method.

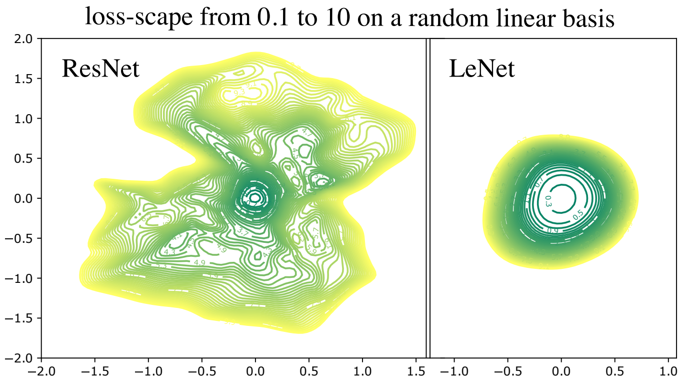

Supplementary Figure D3 Local loss-landscape around a minimum found with a deterministic training on the ResNet (CIFAR-10) and LeNet (EMNIST) task.

Appendix E Derivation details of Metropolis-Hastings with Batch Tempering (MHBT)

This paragraph shortly reviews the two central theorems that guarantee convergence of the proposed GSGHMC algorithm. The first guarantees the preservation of extremal points and the second the convergence to the tempered posterior. While the focus of this paper is the experimental evaluation, these theorems guarantee the convergence of the GSGHMC method. The convergence of HTE is not evaluated, since it will not necessarily converge to a distribution close to the true posterior, but rather a blurry one, that includes the averaging induced by the stochastic gradient. However, as shown by [66], we cannot guarantee the trajectory to stay close to the noise free trajectory and therefore, the bias could be arbitrarily large. We still chose to continue with this method based on our experience with deep neural networks, that usually shows improved generalization when using stochastic gradients instead of gradient descent.

We introduce $\mathcal{X}$ and $\mathcal{Y}$ to be the sets of all possible x and y such that $S \subset (\mathcal{Y}, \mathcal{X})$.
Assumption 1: There exists a function f_1 and a vector of measurable functions $\mathbf{f}$ such that $|\log p(y|x,\boldsymbol{\theta}) - \log p(y'|x',\boldsymbol{\theta})| \leq f_1(\boldsymbol{\theta})||\mathbf{f}(\mathbf{y},\mathbf{x}) - \mathbf{f}(\mathbf{y}',\mathbf{x}')||_\mathbf{1}$, $(y,x), (y',x') \in (\mathcal{Y}, \mathcal{X})$ with $\sup_{\boldsymbol{\theta}\in\boldsymbol{\theta}} f_1(\boldsymbol{\theta}) < \infty$ and $\mathbb{E}_{\boldsymbol{\theta}^*} e^{\delta_1 ||\mathbf{f}(\mathbf{Y},\mathbf{X})}||_1 < \infty$.
The random variables Y, X are defined over $\mathcal{Y}$ and $\mathcal{X}$ and $\mathbb{E}_{\boldsymbol{\theta}^*}$ is the expectation taken over the data generated by the true parameter $\boldsymbol{\theta}^*$.
Assumption 2: There exists a measurable function f_2 such that $|\log p(y|x,\boldsymbol{\theta}) - \log p(y|x,\boldsymbol{\theta}')| \leq f_1(y,x)||\boldsymbol{\theta} - \boldsymbol{\theta}'||_1$ for all $\boldsymbol{\theta}, \boldsymbol{\theta}' \in \boldsymbol{\theta}$ and $(y,x) \in (\mathcal{Y}, \mathcal{X})$. In addition, there exists $\delta_2 > 0$ such that $\mathbb{E}_{\boldsymbol{\theta}^*} e^{\delta_2 f_2(Y,X)} < \infty$.
Assumption 1 and 2 require the log-likelihood to be suitable smooth. This is a common assumption, even in the context of deep learning, although the exact Lipschitz functions are usually impossible to determine. The next assumption is only required for the preservation of extrema theorem and requires well separated extrema.

Assumption 3: $\mu_{\boldsymbol{\theta}} = \mathbb{E}_{\boldsymbol{\theta}^*} \log p(Y, X|\boldsymbol{\theta})$ is twice continuously differentiable in $\boldsymbol{\theta}$. The stationary point of $\mu_{\boldsymbol{\theta}}$, $\boldsymbol{\theta}_0 \in \text{Int}(\boldsymbol{\theta})$, and the Hessian of $\mu_{\boldsymbol{\theta}}$ at $\boldsymbol{\theta}_0$ has eigenvalues $\lambda_i(H_{\boldsymbol{\theta}_0}) < 0 \quad \forall i = 1, \ldots, d$.
Preservation of minima theorem [65]**:** Suppose $\boldsymbol{\theta}_0$ is a stationary point of $\mu_{\boldsymbol{\theta}}$ satisfying Assumption 3. For some $\alpha > 0$, let $c_n \to \infty$ be a sequence such that $\frac{dc_n^{2+\alpha} \log c_n}{m} \to 0$, t be a fixed constant with $t \in (0, \frac{1}{2})$, and $\delta_n = \sqrt{\frac{3\log(1/(1-2t))}{\epsilon_0 c_n}}$. Then under Assumptions 1, 2 for large n,

$$\sup_{\boldsymbol{\theta} \in R_n} \log \tilde{\pi}(\boldsymbol{\theta}) \leq \sup_{\boldsymbol{\theta} \in \mathcal{B}(\boldsymbol{\theta}_0, \delta_n)} \log \tilde{\pi}(\boldsymbol{\theta}) - \log(1/(1-2t)), \tag{E1}$$

with probability at least $1 - \eta_n$. Here $R_n = \{\boldsymbol{\theta} \in \boldsymbol{\theta} : \delta_n < ||\boldsymbol{\theta} - \boldsymbol{\theta}_0||_2 < \delta_0\}$, $\mathcal{B}(\boldsymbol{\theta}_0, \delta_n) = \{\boldsymbol{\theta} \in \boldsymbol{\theta} : ||\boldsymbol{\theta} - \boldsymbol{\theta}_0||_2 < \delta_n\}$, and $\eta_n \leq c_1 \frac{1}{t^2 \lfloor n/m \rfloor c_n^\alpha}, \frac{1}{t^2 \lfloor n/m \rfloor c_n^\alpha} \leq c_2 \eta_n$ for constants
$c_1, c_2 \in \mathbb{R}$.
Assumption 4: $\boldsymbol{\theta}$ is compact.
Convergence theorem [65]**:** Denote $\pi_T(\boldsymbol{\theta}) \propto \pi^{1/T}(\boldsymbol{\theta})$ the tempered posterior. Under Assumptions 1, 2 and 4, for some $\alpha > 0$, $\epsilon_n \to 0$ slower than $c_n^{-\alpha/2}$, $c_n \to \infty$ such that $\frac{dc_n^{2+\alpha} \log c_n}{m} \to 0$, $\mathrm{D}_{KL}(\pi_T || \tilde{\pi}) \leq \epsilon_n$ with probability at least $1 - \eta'_n$, $\eta'_n \leq c_1 \frac{1}{\epsilon_n^2 \lfloor n/m \rfloor c_n^\alpha}, \frac{1}{\epsilon_n^2 \lfloor n/m \rfloor c_n^\alpha} \leq c_2 \eta'_n$ for constants
$c_1, c_2 \in \mathbb{R}$ and large n.

Appendix F Additional information on the greedy subsampling experiment

The selection process used in section 2.5 is realized through the following algorithm 4. It operates on the validation test set and removes samples by the remaining ensemble performance.

The output selection depends on the order of the iteration through the ensemble E. To evaluate the prediction variance caused by this order, we run the algorithm ten times with different initial orders.

To demonstrate the effects of the sample-wise predictive distribution, we choose five different samples with different output distributions and show boxplots of these distributions in figure F4. The samples are for the OBABO SGHMC without acceptance. Here, we observe a reduction of extreme values with the model selection process

Algorithm 4 Greedy ensemble reduction algorithm.

```
SELECT(E, U_val)
for θ_i in E do
    if U_val(E \ {θ_i}) ≤ U_val(E) then
        E = E \ {θ_i}
    end if
end for
```

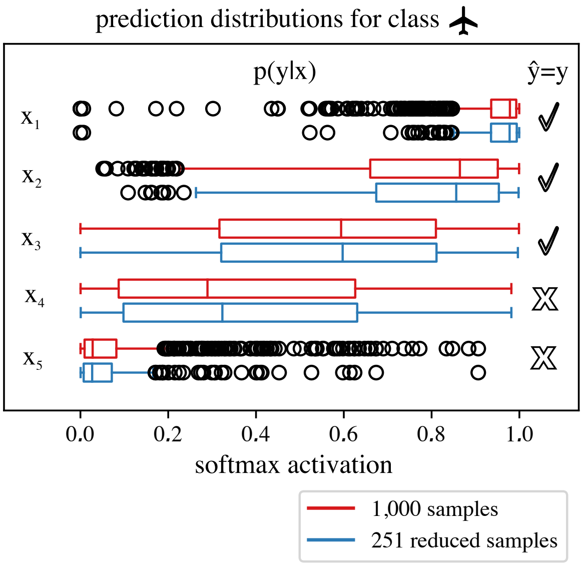

Supplementary Figure F4 Subsampling a Bayesian ensemble created via SGHMC does barely influence the output distribution. The sample distributions are chosen to demonstrate high certainty and high uncertainty input samples. The experiment is the OBABO sampling on the ResNet model (cp. Fig. F4)

and a generally stable predictive distribution on the maximum mean prediction. We chose the examples, such that the five different typical predictions are visible. The one at the top and on the bottom represent the highly certain predictive distributions for wrong and truly classified test samples. The middle represents different predictive distributions in between these confidences. Both the predictive distributions as well as the ensemble accuracy does not show large changes after reducing the ensemble size.

Appendix G On convergence and hyperparameters

The convergence of the algorithms, while theoretically discussed in sections E and 4.5, is further analyzed in this section with an additional experiment. We reduce the main problem of the non-convex likelihood approximated in this paper to a simple double-well potential as this shows the mixing properties of the algorithms and we can also show how well the approximation fits the distribution with the well-known kernel Stein discrepancy (KSD)

$$\exp(-U(\boldsymbol{\theta}))\,\text{with}\,U(\boldsymbol{\theta}) = (\boldsymbol{\theta}+4)(\boldsymbol{\theta}+1)(\boldsymbol{\theta}-1)(\boldsymbol{\theta}-3)/14+0.5. \tag{G2}$$

We run sampling chains with 10 different random seeds starting at the initial value of zero. While this example was also used in [29], we can compare against strong hyperparameters used in the proposing papers. What is shown in figure G5 is the development of the distance between the distribution and its approximation by the accuracy of the predictive mean computed in a Monte Carlo approximation with the samples generated by the sampling algorithms. We observe, that the algorithms do not always get

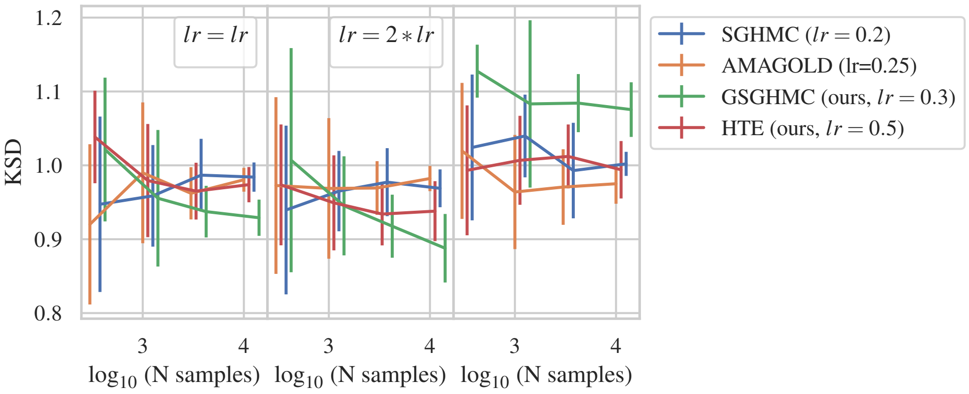

Supplementary Figure G5 The development of approximating a double-well potential with an increasing number of samples. This experiment shows mixing properties of the algorithms depending on the learning rate, which is the most relevant hyperparameter. The distance of distribution approximation is measured using the Kernel Stein Discrepancy, measuring the accuracy of computing the expectation regarding this distribution with an approximating ensemble of samples.

continuously better. Whether the KSD still improves depends on the hyperparameters. Starting with reasonable hyperparameters we observe similar performance of the considered algorithms and slight improvements with GSGHMC. Increasing the step size by factor two shows improvements of both proposed algorithms, although the HTE algorithm does not necessarily converge to the true distribution. As we motivated the algorithms, both show benefits, when exploration is required and both show stable sampling, when the step size is large. AMAGOLD shines with stable behavior when the step size is reduced in the third plot. However, on neural networks exploration with few gradient steps is wanted. For these applications, the two proposed algorithms show promising results, which align to the results presented in the paper.
